# Supplementary material for: A set of multi-entry identification keys to African frugivorous flies (Diptera, Tephritidae)
Source: Zookeys. 2014 Jul 24;(428):97–108. doi: 10.3897/zookeys.428.7366 (PMC4143993; doi:10.3897/zookeys.428.7366)
Supplement: Supplementary material 6 — Key to Ceratitis [file zookeys-428-097-s006.zip › SF6_ZooKeys_key to Ceratitis/key/SF6_ZooKeys_key to Ceratitis/Media/Html/Ceratitis pennitibialis.htm]

Ceratitis (Pterandrus) pennitibialis, n


***Ceratitis*** **(*Pterandrus*) *pennitibialis* De Meyer & Freidberg**

Body
length: 4.28 (3.75-4.70) mm; wing length: 4.67 (4.45-5.00) mm.

Male

Head: Antenna yellow. First flagellomere twice as long as
pedicel. Arista with short rays, basoventral rays shorter and sparser than
basodorsal rays. Frons convex; yellow, with short scattered setulae distinctly
darker than frons. Frontal setae well developed. Face yellowish white. Gena at
most with darker yellow spot. Genal seta black or reddish; genal setulae dark,
poorly developed. Occiput on dorsal part yellowish brown (but not dark brown as
in C. curvata)
except along dorsal margin.

Thorax: Postpronotal
lobe whitish, with brown spot. Scutal pattern; ground color brown, microtrichose
areas silvery with ashgray shine, spots brownish black, prescutellar white or
yellow markings small, separate. Scapular setae dark. Scutellum black, basally
near lateral margin and in median part with yellow spots. Anepisternum on
ventral half yellowish brown; setulae pale (rarely one dark setula ventral to
anepisternal seta); dorsal half with whitish stripe along dorsal margin.

Legs:
Yellow except where otherwise noted; setation typical for subgenus, mixed pale
and dark. Foreleg: femur yellow except dorsally darker, posteriorly with
brownish streak on apical half; anteroventrally with row of very short and
dispersed silvery setulae along entire length (not dense and long comb as in *curvata*);
posteriorly with dispersed mix of pale and dark setulae; posterodorsal row
pale, apically dark, posterior row black along entire length; ventral setae
black. Midleg: femur posteriorly brown except on basal part, brownish in median
part anteriorly, with silvery oblique spot on distal 0.33, silvery shine
conspicuous when viewed from certain angle; ventrally with black feathering on
apical half, continued basally as row of dispersed, and slightly longer
setulae; tibia brown except basally and apically; with dispersed rows of black
feathering dorsally along distal 0.8 and ventrally along distal 0.66;
anteriorly with short setulae, in front of dorsal feathering with row of longer
setulae. Hindleg: femur at apical 0.25 with longer setulae dorsally, less so
ventrally.

Wing:
bands brownish. Marginal band forming continuous band with anterior part
of discal band; cubital band free; medial band absent; crossvein R-M at or just
proximal to middle of discal cell. Apex of vein R_1_ distal to level
of crossvein R-M. Crossvein DM-Cu oblique anterobasally.

Abdomen:
Mostly brownish black. Tergite 1 yellow along anterior margin; tergite 2 across
posterior half to 0.66 and tergite 4 largely with silvery band; tergites 2 and
5 with yellow median patch. Setation and banding typical for subgenus. Male
epandrium in lateral view distinctly broadened; posterior lobe of lateral
surstylus with base broadened, apical end bluntly pointed.

Female

As
male except for the following characters: Antenna yellowish orange; first
flagellomere 2-3 times as long as second. Genal seta dark. Gena with more
distinct brown spot. Ventral half of anepisternum brown with dark setulae on
posterior half. Crossvein DM-Cu oblique posterobasally. Legs without
feathering; yellowish brown except brownish femora; pilosity and ventral setae
of forefemur dark. Oviscape shorter than preabdomen. Aculeus about six times longer
than wide; tip pointed, and lateral margin slightly sinuous.

(Description
after De Meyer & Freidberg, 2006)
